# Exploring the therapeutic potential of *Nelumbo nucifera* leaf extract against amyloid-beta-induced toxicity in the *Caenorhabditis elegans* model of Alzheimer’s disease

**DOI:** 10.3389/fphar.2024.1408031

**Published:** 2024-06-24

**Authors:** Yong-Ping Wen, Hai-Jun Fu, Qin Chen, Cai Lan, Da-Lian Qin, Jian-Ming Wu, An-Guo Wu, Xiao-Gang Zhou

**Affiliations:** ^1^ College of Food and Bioengineering, Chengdu University, Chengdu, China; ^2^ Luzhou Key Laboratory of Activity Screening and Druggability Evaluation for Chinese Materia Medica, Key Laboratory of Medical Electrophysiology of Ministry of Education, School of Pharmacy, Southwest Medical University, Luzhou, China; ^3^ Nanchong Food and Drug Inspection Institute, Nanchong, China; ^4^ Central Nervous System Drug Key Laboratory of Sichuan Province, Luzhou, China

**Keywords:** Alzheimer’s disease, Aβ, *Nelumbo nucifera* leaf extract, *Caenorhabditis elegans*, autophagy

## Abstract

**Introduction:** Alzheimer’s disease (AD) represents a critical global health challenge with limited therapeutic options, prompting the exploration of alternative strategies. A key pathology in AD involves amyloid beta (Aβ) aggregation, and targeting both Aβ aggregation and oxidative stress is crucial for effective intervention. Natural compounds from medicinal and food sources have emerged as potential preventive and therapeutic agents, with *Nelumbo nucifera* leaf extract (NLE) showing promising properties.

**Methods:** In this study, we utilized transgenic *Caenorhabditis elegans* (*C. elegans*) models to investigate the potential of NLE in countering AD and to elucidate the underlying mechanisms. Various assays were employed to assess paralysis rates, food-searching capabilities, Aβ aggregate accumulation, oxidative stress, lifespan under stress conditions, and the expression of stress-resistance-related proteins. Additionally, autophagy induction was evaluated by measuring P62 levels and the formation of LGG-1^+^ structures, with RNAi-mediated inhibition of autophagy-related genes to confirm the mechanisms involved.

**Results:** The results demonstrated that NLE significantly reduced paralysis rates in CL4176 and CL2006 worms while enhancing food-searching capabilities in CL2355 worms. NLE also attenuated Aβ aggregate accumulation and mitigated Aβ-induced oxidative stress in *C. elegans*. Furthermore, NLE extended the lifespan of worms under oxidative and thermal stress conditions, while concurrently increasing the expression of stress-resistance-related proteins, including SOD-3, GST-4, HSP-4, and HSP-6. Moreover, NLE induced autophagy in *C. elegans*, as evidenced by reduced P62 levels in BC12921 worms and the formation of LGG-1^+^ structures in DA2123 worms. The RNAi-mediated inhibition of autophagy-related genes, such as *bec-1* and *vps-34*, negated the protective effects of NLE against Aβ-induced paralysis and aggregate accumulation.

**Discussion:** These findings suggest that NLE ameliorates Aβ-induced toxicity by activating autophagy in *C. elegans*. The study underscores the potential of NLE as a promising candidate for further investigation in AD management, offering multifaceted approaches to mitigate AD-related pathology and stress-related challenges.

## 1 Introduction

Alzheimer’s disease (AD) is a significant global health concern impacting a large number of people and imposing substantial burdens on healthcare systems. Beyond imparting profound emotional distress on the families, AD also demands extensive financial resources for management and treatment (Association, 2018). Disturbingly, someone around the globe is diagnosed with AD once every 3 s. Predictions estimate that by 2030 and 2050, the numbers of dementia cases could reach 82 and 152 million, respectively, with AD comprising a staggering 60%–70% of these cases (Sharma and Singh, 2023). Regrettably, the current therapeutic options for AD are scarce, and there are only six FDA-approved medications whose efficacies are controversial, while some are even associated with considerable side effects (Dunn et al., 2021). Consequently, an intensive exploration of safer and more potent anti-AD strategies or agents that are harmonized with the specificities of AD is crucial for managing this debilitating disease.

Although the intricate mechanisms underlying AD remain elusive, the hallmark pathologies of AD, especially amyloid beta (Aβ) aggregation plaques and tau neurofibrillary tangles, are well-established (Ana et al., 2023). Research indicates that Aβ aggregation contributes to mitochondrial dysfunction and oxidative stress, which further amplify cellular dysfunction and accelerate the progression of AD by reinforcing Aβ aggregation (Rehman et al., 2023; Alqahtani et al., 2023). Given these insights, strategies aimed at mitigating Aβ aggregation and neutralizing the resulting oxidative stress appear to be pivotal in the intervention of AD. Natural compounds, particularly those with medicinal and food properties, are gaining prominence as prospective preventive and therapeutic agents for neurodegenerative diseases like AD owing to their diverse components and targets, notable safety profiles, and minimal clinical side effects (Atlante et al., 2020). For instance, *Luffa cylindrica* fruit extracts have been proven to alleviate AD-associated pathologies by stimulating autophagy in various *Caenorhabditis elegans* and cellular models (Long et al., 2023a; Iranshahy and Javadi, 2019). Additionally, *Ligusticum chuanxiong* Hort that is recognized as both a medicinal and an edible plant has exhibited potent antiaging, antioxidant, and neuroprotective properties in *C. elegans* (Qin et al., 2022). Olfactory stimulation by essential oils originating from both edible and medicinal homologous plants has demonstrated improvements in Aβ-induced cognitive deficits by modulating oxidative stress and synaptic functions (Wei et al., 2023). Nevertheless, the currently identified medicinal and edible homologous plants do not address clinical requirements sufficiently. Hence, the exploration of additional compounds that share origins in both medicine and food could proffer more alternatives in addressing the challenges posed by AD.

*Nelumbo nucifera* Gaertn., commonly known as the sacred lotus and belonging to the Nymphaeaceae family, has been cherished globally for its dual role as both a functional food and medicinal herb, especially in Asian traditions dating back to ancient times (Mukherjee et al., 2009). Within this plant, the *Nelumbo nucifera* leaf holds a prominent position and has been celebrated in local cultures for its diverse range of medicinal properties encompassing diuretic, cardiotonic, and vasodilatory functions (Zhang et al., 2022). Historically, this plant has played pivotal roles in remedying health challenges, such as obesity, diarrhea, insomnia, hyperglycemia, and specific skin disorders (Chen G. et al., 2019). Beyond these traditional uses, modern research has shown that the vast pharmacological potential of *N. nucifera* leaves, showcasing their antioxidant, anti-inflammatory, immunomodulatory, hepatoprotective, and antiviral virtues (Wang et al., 2022). Despite these attributes, the specific impacts and intrinsic mechanisms of *N. nucifera* leaves in addressing AD-related conditions are not fully known.

Recently, *C. elegans* has gained popularity as an invaluable model in pathological studies, particularly those related to aging and associated diseases, owing to its simplistic nervous architecture, transparent genetic background, brief lifecycle, and straightforward phenotype analysis (Van Pelt and Truttmann, 2020). Significantly, the *C. elegans* genome shares a remarkable similarity to that of humans, making it an apt *in vivo* model for AD research because its neuron functionalities and neurotransmitters resemble those of more complex organisms (Godini et al., 2019). In the present research, transgenic *C. elegans* models were used to evaluate the therapeutic potential of *N. nucifera* leaf extract (NLE) against AD. Our results showed that NLE significantly reduced Aβ-induced paralysis and hindered detrimental Aβ aggregation. Mechanistic studies were conducted to demonstrate that NLE induced autophagy in worms, while the RNAi-mediated inhibition of autophagy-related genes abrogated the protective effects of NLE against Aβ-induced toxicity and aggregate accumulation. These compelling observations are expected to lay a robust theoretical foundation, suggesting that *N. nucifera* leaves may hold substantial promise as potent therapeutic agents in the battle against AD.

## 2 Materials and methods

### 2.1 Preparation of NLE

*N. nucifera* leaves were procured from Yongyi TCM Co., Ltd. (Hebei, China) and deposited in Southwest Medical University with a voucher specimen (No. SWMU-VS202211). To obtain the ethanolic extract of *N. nucifera* leaves, approximately 100 g of the leaves was meticulously chopped and then pulverized to achieve a particle size ranging from 40 to 60 mesh. The resulting powder was subjected to extraction thrice using 95% ethanol (500 mL each time) for a total duration of 2.5 h at 65°C. The resultant liquids from these extractions were amalgamated, sifted, and then concentrated in vacuum using a rotary evaporator. This crude concentrate was later redissolved in dimethylsulfoxide (DMSO) for further analysis. All subsequent experimental solutions were prepared by diluting this stock after ensuring that the final DMSO concentration was below 0.1%.

### 2.2 UHPLC-DAD-Q/TOF-MS/MS conditions

The chemical composition analysis of NLE involved an ultrahigh-performance liquid chromatography system equipped with diode array detection (Shimadzu, Kyoto, Japan) and coupled with quadrupole time-of-flight tandem mass spectrometry (UHPLC-DAD-Q/TOF-MS/MS). Separation of NLE component was performed on an Agilent Zorbax EcLipse Plus C18 column (1.8 μm, 100 mm × 2.1 mm) at a flow rate of 0.3 mL/min, with the column maintained at 40°C. The mobile phase comprising eluent A (0.1% formic acid in water, v/v) and eluent B (0.1% formic acid in acetonitrile, v/v) was obtained in accordance with a previously established methodology (Pei et al., 2021), where the electrospray ionization (ESI) parameters were set in accordance with prior specifications (Pei et al., 2021). Subsequent data analysis was performed using Peak View^®^ 1.4 software (AB SCIEX, Foster City, CA, United States).

### 2.3 *C. elegans*: acquisition, culture, and synchronization

To assess the impacts of NLE on Aβ-induced pathology, antioxidant capacity, and autophagy activation in worms, multiple *C. elegans* strains were used in the current study, namely Bristol N2, CL802 [*rol-6*(su1006)] II, CL4176 dvIs27 [Pmyo-3:human A-beta 1–42; let-851 3′UTR; *rol-6*(su1006)], CL2006 dvIs2 [Punc-54:human A-beta 3–42; pRF4 *rol-6*(su1006)], CL2331 dvIs37 [myo-3p:GFP::A-beta (3–42) + *rol-6*(su1006)], CL2355 pCL45 [Psnb-1:human A-beta 1–42:3′UTR (long); Pmtl-2:GFP], CL2122 dvIs15 [(pPD30.38) unc-54(vector) + (pCL26) mtl-2:GFP], SJ4100 zcIs13[hsp-6:GFP], SJ4005 zcIs4 [hsp-4:GFP] V, CL2166 dvIs19 [(pAF15)gst-4p:GFP::NLS] III, CF1553 muIs84 [(pAD76) sod-3p:GFP + *rol-6*(su1006)], DA2123 adIs2122 [lgg-1p:GFP:lgg-1 + *rol-6*(su1006)], and BC12921 sIs10729 [rCes T12G3.1:GFP + pCeh361]. All these strains were procured from the *Caenorhabditis* Genetics Center (CGC, University of Minnesota, Minneapolis, MN, United States). The worms were then cultured at 20°C, except for CL4176, CL2331, and CL2355, which were grown at 15°C on nematode growth medium (NGM) agar plates supplemented with *Escherichia coli* (either OP50 or transformed HT115). To achieve synchronized L1 larvae, the embryos were isolated via a bleaching solution and subsequently incubated in M9 buffer. Post incubation, the L4 larvae were placed on NGM plates infused with 5 mg/L 5-fluoro-2′-deoxyuridin (FUDR, sourced from Sigma) to prevent offspring hatching.

### 2.4 Paralysis assessments and rolling movement analyses in CL4176 and CL2006 strains

Paralysis assessments were performed with the CL4176 and CL2006 worms based on methodologies reported in a previous study (Long et al., 2023b). Initially, synchronized L1 larvae of the CL4176 worms were cultured on NGM plates at 15°C for 36 h. Then, they were transferred to a 25°C setting to trigger accelerated Aβ_(1–42)_ protein expression culminating in paralysis. On the contrary, the CL2006 larvae were raised at 20°C until they attained the late L4 stage. Thereafter, they were relocated to fresh NGM plates containing 5 mg/L FUDR to prevent egg hatching. For paralysis detection, a platinum wire was utilized to gently tap the worm heads. Worms with mobile heads but immobile bodies were designated as “paralyzed.” Each experiment necessitated a minimum of 50 worms per group, and the paralysis assessments were performed at least three times. For the rolling movement assay of the CL4176 worms, the nematodes were subjected to treatment with or without NLE for a duration of 24 h. Subsequently, the numbers of rolls made by the nematodes were counted over a period of 20 s. Each experiment required 15 worms per group, and the assay was conducted at least three times.

### 2.5 Evaluation of Aβ_(3–42)_ aggregation in the CL2331 strain

To assess the impact of NLE on Aβ aggregation in worms, the CL2331 strain that constitutively expresses the Aβ_(3–42)_-GFP fusion protein in its body wall muscle was utilized. This continuous expression of Aβ_(3–42)_-GFP enables real-time visualization of Aβ aggregation *in vivo*. Initially, the synchronized L1 larvae were treated with NLE at 15°C until they progressed to the late L4 stage. Thereafter, they were shifted to a 25°C environment and exposed to NLE for an additional 48 h. Upon treatment completion, these worms were harvested using M9 buffer and positioned onto slides equipped with 2% agarose foundation. Fluorescent images of the nematodes were then captured using a positive fluorescence microscope (Leica DM6B, Leica Microsystems GmbH, Germany), and the images were magnified to count the Aβ_(3–42)_ aggregates within the worms. Each experiment involved a minimum of 20 worms per group, and the aggregation assessments were performed at least three times.

### 2.6 Food-sensing behavior examination

The food-sensing behavior assay was performed to assess the effects of Aβ on the functions of neurons in *C. elegans*. Initially, the synchronized L1 larvae of the CL2355 worms that express the human Aβ_(1–42)_ protein throughout their neurons and control worms (CL2122) were maintained at 15°C for 36 h. Thereafter, the temperature was increased to 25°C, and the worms were further incubated for 36 h. Subsequently, the worms were repositioned centrally on NGM agar plates that either contained or lacked *E. coli* OP50. After 2 min, their locomotion rates were noted in 20-s intervals. A specific formula was applied to compute the slowing rate as (N _without food_–N _with food_)/N _without food_, where N indicates the total body bends in the presence or absence of bacteria. Each experiment involved 20 worms per group, and the behavior examinations were conducted at least thrice.

### 2.7 Stress resistance assessments in *C. elegans*

For the thermal tolerance assays, synchronized L1 larvae of the N2 worms were shifted to fresh NGM plates with or without NLE until they reached the late L4 stage. Then, the worms were moved to new NGM plates containing FUDR to prevent offspring hatching and were maintained at 20°C for 5 days. After this treatment, the environmental temperature was increased to 35°C, and the non-responsive worms (assessed through platinum wire touch) were recorded hourly. For the oxidative stress resistance experiments, the synchronized L1 larvae were initially placed on fresh NGM plates with NLE and allowed to grow at 20°C until they reached the late L4 stage. They were then transferred to new NGM plates containing 50 mM H_2_O_2_ to induce acute oxidative stress. Hourly assessments were then conducted, with the worms unresponsive to platinum wire touch being classified as deceased. At least 50 worms per group were required in each experiment, and each of these tests was replicated thrice. The survival patterns were analyzed using the Kaplan–Meier approach, while the statistical relevance was evaluated using the log-rank test by employing GraphPad Prism 9.0 toolkit.

### 2.8 Assessments of reactive oxygen species (ROS) levels and activities of superoxide dismutase (SOD), glutathione peroxidase (GSH-Px), and catalase (CAT) in *C. elegans*

To quantify the ROS levels in the worms, NLE-treated CL4176 or CL802 (control worms) were gathered and exposed to 100 μM dihydroethidium (DHE) staining in a 500 μL solution for 1 h at 20°C. After immobilization with 10 mM sodium azide, the worms were mounted on glass slides using 2% agarose pads. Fluorescent images of the worms were then captured using a positive fluorescence microscope, and ImageJ software was employed to measure the red fluorescence intensity to serve as an indicator of ROS level. This quantification involved measuring the intensities of the segmented regions of interest in the worms and normalizing them with respect to the body area to calculate the average pixel intensity. Each experiment was performed in triplicate, and 15 worms were assessed in each replication. To evaluate the SOD, GSH-Px, and CAT activities in the worms, NLE-treated CL4176 worms were collected, ground in phosphate-buffered saline (PBS), and centrifuged to collect the supernatant. The supernatant was then used to assay the SOD, GSH-Px, and CAT activities with commercial test kits from Beyotime Biotechnology in China as per manufacturer instructions. Each experiment was performed in triplicate to ensure the robustness and reliability of the results.

### 2.9 Fluorescence intensity quantification assay

The fluorescence intensity quantification assay was conducted to assess the impacts of NLE on the expressions of various proteins, including *hsp-4*::GFP, *hsp-6*::GFP, *sod-3*p::GFP, and *gst-4*p::GFP in the transgenic worms SJ4005, SJ4100, CF1553, and CL2166, respectively. In brief, the synchronized L1 worms were transferred to NGM plates containing 200 μg/mL NLE and incubated at 20°C for 2 days. Subsequently, the treated worms were collected in M9 buffer and placed on agarose pad slides with 10 mM sodium azide. Representative images of the worms were captured using a positive fluorescence microscope, and the fluorescence intensity was quantified using ImageJ software by measuring the intensities of the segmented regions of interest in the worms and normalizing them to the body areas to calculate the average pixel intensities. Each experiment involved 15 worms per group, and the assay was repeated for at least three independent experiments.

### 2.10 Evaluation of autophagy in *C. elegans*

The impacts of NLE on autophagy initiation were evaluated in the BC12921 and DA2123 strains by monitoring the green fluorescence protein (GFP) expressions associated with p62/SQST-1-GFP and quantifying the GFP-tagged LGG-1/Atg8 puncta in the worms in accordance with established protocols (He et al., 2023). In brief, BC12921 and DA2123 worms were cultured on NGM plates with or without NLE or rapamycin (Rap, used as a positive control) at 20°C for 48 h. After treatment, the worms were collected in M9 buffer and placed on glass slides with 2% agarose pads to capture representative images using a fluorescence microscope. The fluorescence intensity representing p62 expression in the BC12921 strain was determined by measuring the intensities within the segmented regions of interest in the worms and normalizing them with respect to the body area to calculate the average pixel intensity. GFP::LGG-1/Atg8 puncta quantification in the DA2123 strain was conducted by direct counting. Each group in every experiment consisted of at least 20 worms, and the experiments were replicated thrice independently. To ascertain the protein levels of p62/SQST-1-GFP and ratios of phosphatidylethanolamine (PE) conjugated GFP::LGG-1 (PE-GFP::LGG-1) to non-lipidated GFP::LGG-1 in *C. elegans*, the BC12921 and DA2123 worms were subjected to 48 h of treatment with NLE. Subsequently, the worms were collected using M9 buffer and employed in a Western blotting assay following established protocols from previous studies (Chen Y.-L. et al., 2019).

### 2.11 Implementation of RNAi in *C. elegans*

The RNAi assays were performed by exposing the worms to bacteria expressing RNAs targeting specific genes, consistent with established methodologies (Grishok et al., 2000). In brief, RNAi bacteria of *bec-1* and *vps-34* genes as well as control bacteria HT115 with the empty L4440 vector were cultured overnight at 37°C in Luria–Bertani liquid supplemented with ampicillin (100 mg/L). Subsequently, the bacteria were collected and spread onto NGM plates containing 1 mM isopropyl β-D-1-thiogalactopyranoside (IPTG) to induce double-stranded RNA synthesis. The synchronized L1 larvae were then placed on the IPTG-treated NGM plates infused with either NLE or DMSO. The RNAi clones targeting *bec-1* and *vps-34* were obtained from Ahringer’s RNAi library, and the interference effects of these target genes in *C. elegans* have been elucidated in a previous study (Zhu et al., 2023).

### 2.12 Statistical assessment

Statistical evaluations were performed on data collated from at least three separate experiments. For evaluating the paralysis frequency and stress resilience, the Kaplan–Meier survival curves were employed. The significances in these contexts were determined by the log-rank test. The other data sets are presented in terms of mean values and their standard deviations (SDs). The *p*-values were analyzed by the two-tailed *t*-test. All statistical analyses were conducted using GraphPad Prism version 9.0, where *p*-values less than 0.05 were considered statistically significant.

## 3 Results

### 3.1 UHPLC-DAD-Q/TOF-MS/MS analysis of NLE

**FIGURE 1:**
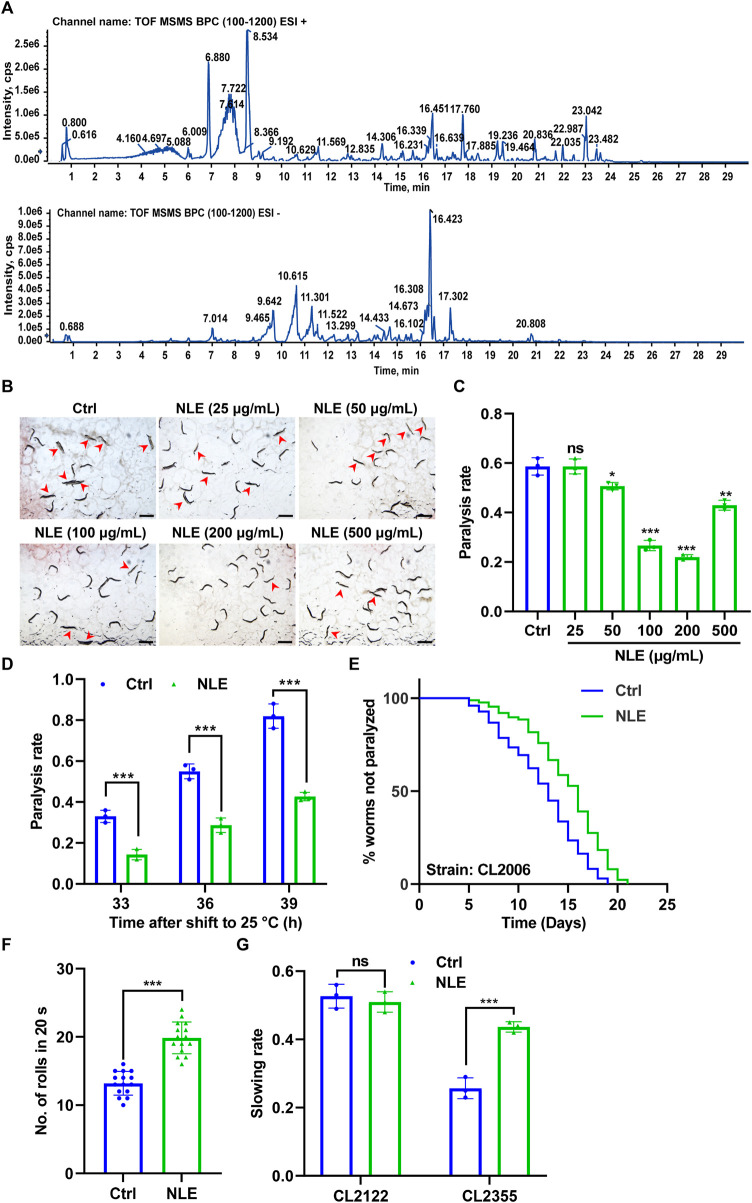
NLE mitigates Aβ-induced toxicity in *Caenorhabditis elegans*. **(A)** UHPLC-DAD-MS chromatogram of NLE. Base peak chromatograms (BPCs) of NLE are shown in the positive (upper panel) and negative (bottom panel) ion modes. **(B)** Representative images showing the paralysis of CL4176 worms treated with various concentrations of NLE (25–500 μg/mL) upon transfer to 25°C for 36 h. Scale bars: 1 mm. **(C)** Bar graph illustrating the paralysis rates of CL4176 worms in **(A)**. Data are presented as mean ± SD (n = 3 independent experiments). **(D)** Bar graphs indicating the paralysis rates of CL4176 worms treated with 200 μg/mL NLE after being transferred to 25°C at the end of 33 h, 36 h, and 39 h. Data are presented as mean ± SD (n = 3 independent experiments). **(E)** Line graphs showing the paralysis rates of CL2006 worms treated with 200 μg/mL of NLE. **(F)** Bar graphs illustrating the rolling numbers of CL4176 worms after 24 h of treatment with or without 200 μg/mL of NLE. Data are presented as mean ± SD (n = 15 nematodes per group). **(G)** Bar graphs indicating the slowing rates of CL2122 and CL2355 worms. Data are presented as mean ± SD (n = 3 independent experiments). For the survival curve statistical analysis, data were analyzed using the Kaplan–Meier method, and the *p*-values were calculated using the log-rank test. For other statistical analyses, *p*-values were calculated using a two-tailed *t*-test. ns *p* > 0.05, *p ≤ 0.05, **p ≤ 0.01, and ***p ≤ 0.001. *p* < 0.05 was considered to be statistically significant.

In this study, we characterized the bioactive constituents of NLE using UHPLC-DAD-Q/TOF-MS/MS to ensure sample quality. Representative base peak chromatograms (BPCs) of the NLE in the positive and negative ion modes for this analysis are depicted in Figure 1A. A comprehensive list of the components identified in NLE, including their chemical names, molecular formulas, masses, and retention times, is presented in Table 1. The primary constituents encompass a diverse array of alkaloids, flavonoids, phenols, and other substances, such as nuciferine, leonticine, dauriciline, sinomenine, chrysoeriol, and kaempferol. These findings are aligned with the components identified in a previous study (Pei et al., 2021).

**TABLE 1:** Chemical names, retention times, chemical formulas, and masses of the components identified in the *Nelumbo nucifera* leaf extract.

Chemical name	Retention time (min)	Chemical formula	Expected mass (Da)	Observed mass (Da)	Mass error (Da)
Leonticine	11.80	C_17_H_19_NO_3_	327.183	327.1827	0.0003
Dauriciline	22.95	C_36_H_40_N_2_O_6_	596.2886	596.2841	0.0045
Sinomenine	6.68	C_19_H_23_NO_4_	329.1627	329.1626	0.0001
Nuciferine	20.83	C_19_H_21_NO_2_	295.1572	295.1573	0.0001
Kaempferol	5.81	C_15_H_10_O_6_	286.0477	286.0465	0.0012
Floribundine	5.07	C_18_H_19_NO_2_	281.1416	281.1403	0.0013
Glucoobtusifolin	8.22	C_22_H_22_O_10_	446.1213	446.1265	0.0052
1,3-Dicaffeoylquinic acid	7.11	C_25_H_24_O_12_	516.1268	516.1270	0.0002
Loliolide	18.47	C_11_H_16_O_3_	196.1099	196.1088	0.0011
Lysicamine	8.87	C_18_H_13_NO_3_	291.0895	291.0837	0.0058
Chrysoeriol	8.05	C_16_H_12_O_6_	300.0634	300.0611	0.0023
Gentiotriose	10.23	C_18_H_32_O_16_	504.169	504.1675	0.0015
Gentiatibetine	12.90	C_9_H_11_NO_2_	165.079	165.0737	0.0053
Coclaurine	10.55	C_17_H_19_NO_3_	271.1208	271.1299	0.0091
Armepavine	6.90	C_19_H_23_NO_3_	313.1678	313.1670	0.0008
N-Methylisococlaurine	7.66	C_18_H_21_NO_3_	299.1521	299.1555	0.0034
Esculetin	8.40	C_9_H_6_O_4_	178.0266	178.0284	0.0018
Cnidimol D	8.10	C_15_H_16_O_6_	292.0947	292.0960	0.0013
Mongolicumin A	1.64	C_18_H_10_O_8_	354.0376	354.0395	0.0019
Linocinnamarin	11.02	C_16_H_20_O_8_	340.1158	340.1171	0.0013
Phellibaumin A	11.98	C_17_H_14_O_7_	330.074	330.0727	0.0013

### 3.2 NLE alleviates Aβ-induced toxicity in *C. elegans* models of AD

To evaluate the potential of NLE in mitigating Aβ-induced toxicity, the transgenic *C. elegans* strain CL4176 was employed. This strain features temperature-sensitive expression of the human Aβ_(1–42)_ protein in its body wall muscle cells under the control of the muscle-specific *unc-54* promoter, resulting in the formation of toxic aggregates and progressive paralysis (Wu et al., 2006). Following treatments with various concentrations of NLE from 25 μg/mL to 500 μg/mL, the paralysis rates of these worms were assessed. The findings depicted in Figures 1B,C reveal that a concentration of 200 μg/mL of NLE was most effective in reducing paralysis. Consequently, this concentration was chosen for subsequent anti-AD studies. Additionally, the NLE treatment showed decreased paralysis rates in the CL4176 worms at multiple time points (33 h, 36 h, and 39 h) after shifting the temperature to 25°C (Figure 1D). To further substantiate these findings, another transgenic strain CL2006 characterized by continuous Aβ_(1–42)_ expression was utilized. The paralysis assay revealed that NLE supplementation decelerated the progression of paralysis in the CL2006 worms (Figure 1E). Recognizing the potential negative impact of Aβ accumulation on muscle function, the physical effects of NLE on the CL4176 worms were assessed by quantifying the number of rolls. The CL4176 strain featuring a rolling coinjected marker results in rolling of the transgenic nematodes instead of sinusoidal movements. The rolling movement assay of CL4176 demonstrated that NLE treatment enhanced mobility compared to untreated worms (Figure 1F), as evidenced by the increased number of rolls in an interval of 20 s, indicating its potential to alleviate Aβ-induced motor deficits in *C. elegans*. Furthermore, the impact of NLE on neurotoxicity induced by the Aβ protein was investigated. In the transgenic strain CL2355 expressing the human Aβ_(1–42)_ protein throughout the neurons (DanQing et al., 2021), it was observed that the worms faced challenges in food searching relative to the control strain CL2122. However, NLE administration significantly improved their food-searching capabilities, as indicated by the recovered slowing rate (Figure 1G). Overall, these results demonstrate that NLE can counteract Aβ-induced toxicity in *C. elegans*.

### 3.3 NLE decreases Aβ aggregation and reduces Aβ-induced oxidative stress in *C. elegans*

**FIGURE 2:**
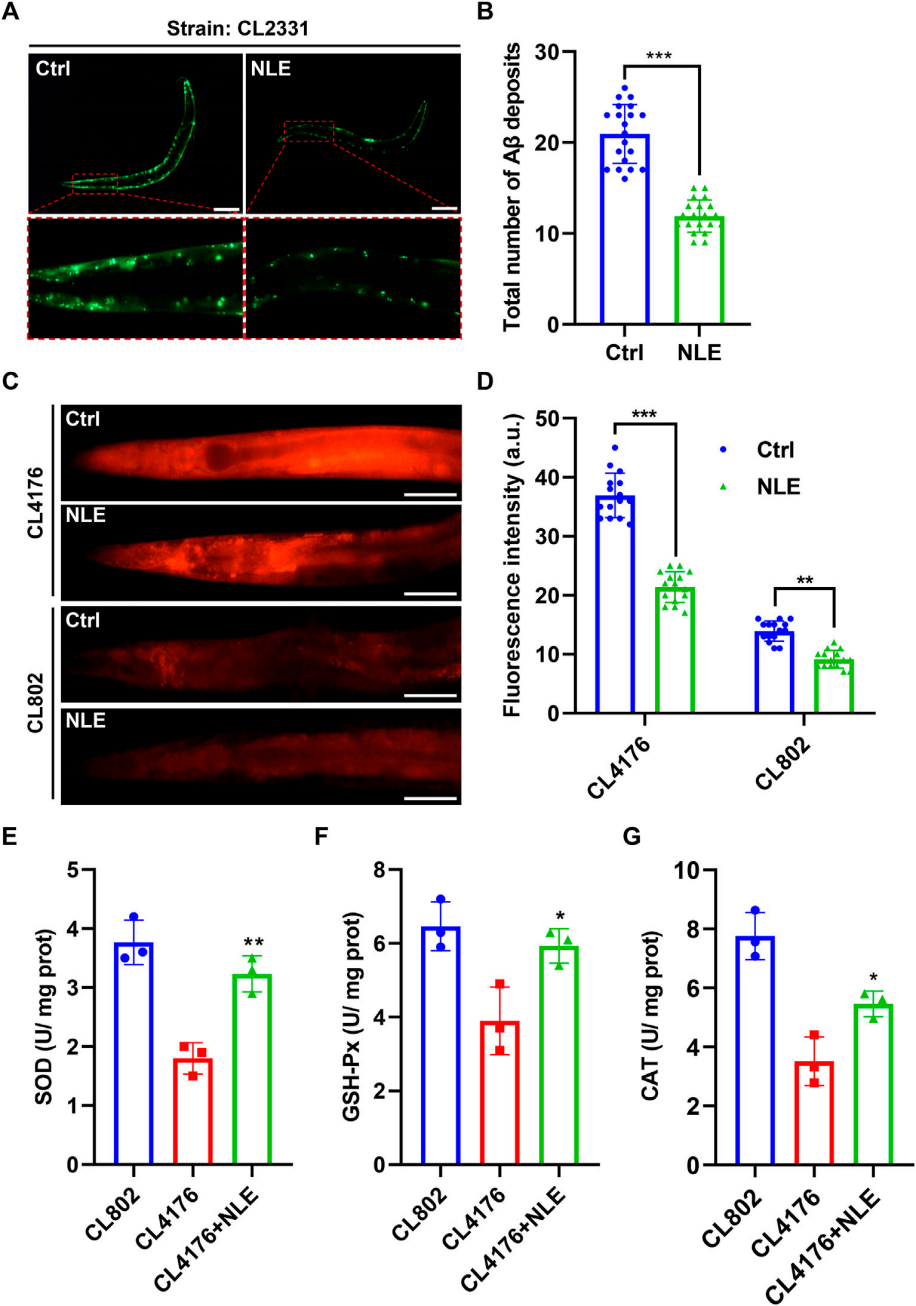
NLE reduces Aβ accumulation and ROS levels in *Caenorhabditis elegans*. **(A,B)** Representative fluorescent images illustrating the accumulation of Aβ deposits in CL2331 worms, with the magnified images highlighting Aβ deposits in the anterior head regions of the CL2331 worms. Scale bars: 200 μm. The bar graph quantifies the total number of Aβ deposits in CL2331 worms. The data are presented as mean ± SD (n = 20 nematodes per group). **(C,D)** Representative fluorescent images and bar graphs showing ROS accumulation in the CL4176 and CL802 worms stained with DHE in the head to mid-trunk region. Data are presented as mean ± SD (n = 15 nematodes per group). **(E–G)** Bar graphs indicating the activities of SOD, GSH-Px, and CAT in NLE-treated CL4176 worms. The data are presented as mean ± SD (n = 3 independent experiments). Scale bars: 50 μm. For statistical analysis, the *p*-values were calculated using a two-tailed *t*-test. **p ≤ 0.01, ***p ≤ .01, and **p < 0.05 were considered statistically significant.

Previous studies have emphasized that the early accumulation of Aβ aggregates in the brain are precursors to AD and that these often manifest years before the emergence of distinct clinical symptoms (Hampel et al., 2021). This study investigates the impacts of NLE on Aβ accumulation in *C. elegans*. Specifically, the CL2331 strain was utilized as it expresses the human Aβ_(3–42)_ protein combined with GFP in a temperature-sensitive manner in its muscle cells. Fluorescence analysis showed that there was a significant reduction in Aβ aggregates in the NLE-treated CL2331 worms compared to the untreated worms (Figures 2A,B), suggesting the effectiveness of NLE in obstructing *in vivo* Aβ aggregation. Abnormal Aβ aggregates in AD have been linked to mitochondrial dysfunction, subsequently leading to oxidative stress and neuronal cell death (Eckert et al., 2003). To explore NLE’s protective properties, the DHE staining method was employed to quantify the ROS levels stemming from the Aβ aggregates. The data illustrated in Figures 2C,D indicate increased ROS generation in the CL4176 worms compared to the wild-type strain CL802, probably owing to Aβ aggregation. However, NLE treatment noticeably reduced the ROS levels in both worm strains. Additionally, the activities of key antioxidant enzymes, including SOD, GSH-Px, and CAT, were diminished in the CL4176 worms than the CL802 worms but were restored upon NLE treatment (Figures 2E–G). These findings collectively underscore the dual capability of NLE to inhibit Aβ aggregate formation and mitigate oxidative stress induced by these aggregates in *C. elegans*.

### 3.4 NLE augments stress resilience in *C. elegans*

**FIGURE 3:**
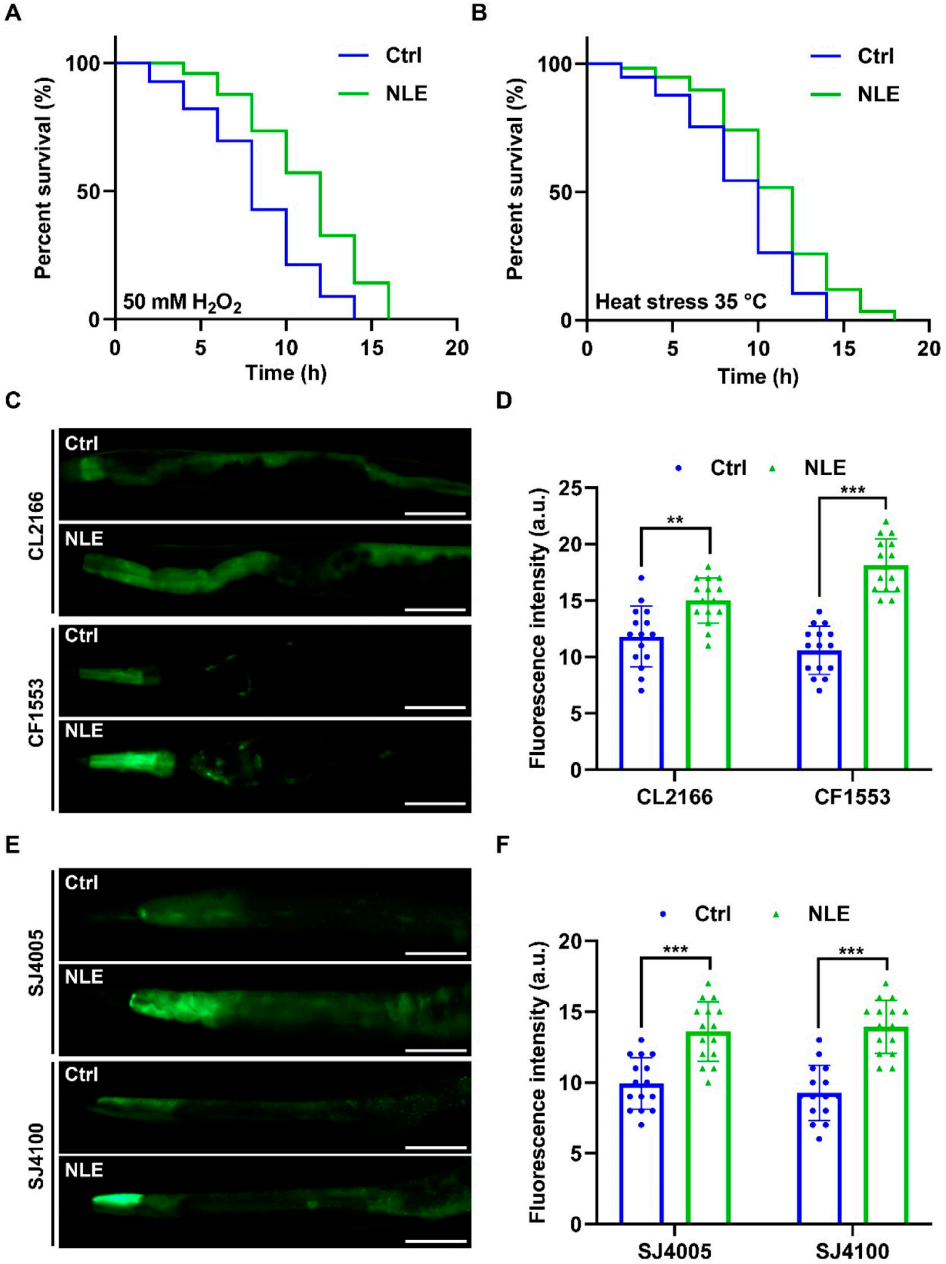
NLE enhances stress resistance in *Caenorhabditis elegans*. **(A)** The line graphs illustrate the survival times of NLE-treated wild-type N2 worms in the presence and absence of 50 mM H_2_O_2_. **(B)** The line graphs illustrate the survival times of wild-type N2 worms treated with NLE at 35°C. **(C,D)** Representative fluorescent images and bar graphs depicting the intensities of *gst-4*::gfp in CL2166 worms and *sod-3*::gfp in CF1553 worms in the head to mid-trunk region. Data are presented as mean ± SD (n = 15 nematodes per group). Scale bars: 50 μm. **(E,F)** Representative photographs and bar graphs representing the expressions of *hsp-4*::GFP in transgenic SJ4005 worms and *hsp-6*::GFP in SJ4100 worms in the head to mid-trunk region. Data are presented as mean ± SD (n = 15 nematodes per group). Scale bars: 50 µm. For the survival curve statistical analysis, data were analyzed using the Kaplan–Meier method, and the *p*-values were calculated using the log-rank test. For other statistical analyses, the *p*-values were calculated using a two-tailed *t*-test. **p ≤ 0.01, ***p 0.01, and ***p < 0.05 were considered to be statistically significant.

As organisms age, their ability to withstand external stressors diminishes, exacerbating the aging process (Long et al., 2022). To investigate whether NLE enhances stress resistance in *C. elegans*, experiments involving oxidative and heat stresses were conducted. In the oxidative stress assay, N2 worms underwent a pretreatment phase with NLE for 2 days at 20°C, followed by exposure to fresh NGM plates containing 50 mM of H_2_O_2_ to induce acute oxidative stress. Remarkably, the application of NLE resulted in a substantial extension of the lifespans of the worms, as evidenced in Figure 3A. Additionally, NLE treatment incited elevated expressions of *sod-3*p::GFP and *gst-4*p::GFP fusion proteins in transgenic strains CF1553 and CL2166, respectively (Figures 3C,D). These proteins are well-recognized markers associated with oxidative stress resistance in *C. elegans*. In the heat stress assay, N2 worms were pretreated with NLE for 5 days at 20°C before being exposed to a temperature of 35°C. As illustrated in Figure 3B, NLE treatment significantly increased the survival rates of the worms subjected to thermal stress. To determine the underlying mechanisms, an examination was conducted on whether NLE influenced the expression of heat shock proteins (HSPs) that are known for their close association with heat stress resistance (Jacob et al., 2017). The findings revealed that NLE treatment substantially upregulated the expressions of HSP-4:GFP and HSP-6:GFP fusion proteins in transgenic strains SJ4005 and SJ4100, respectively (Figures 3E,F). Collectively, the results provide compelling evidence that NLE treatment fortifies the resistance of *C. elegans* to both oxidative and thermal stressors, indicating its potential as an enhancer of stress resilience in this model organism.

### 3.5 NLE facilitates activation of autophagy in *C. elegans*

**FIGURE 4:**
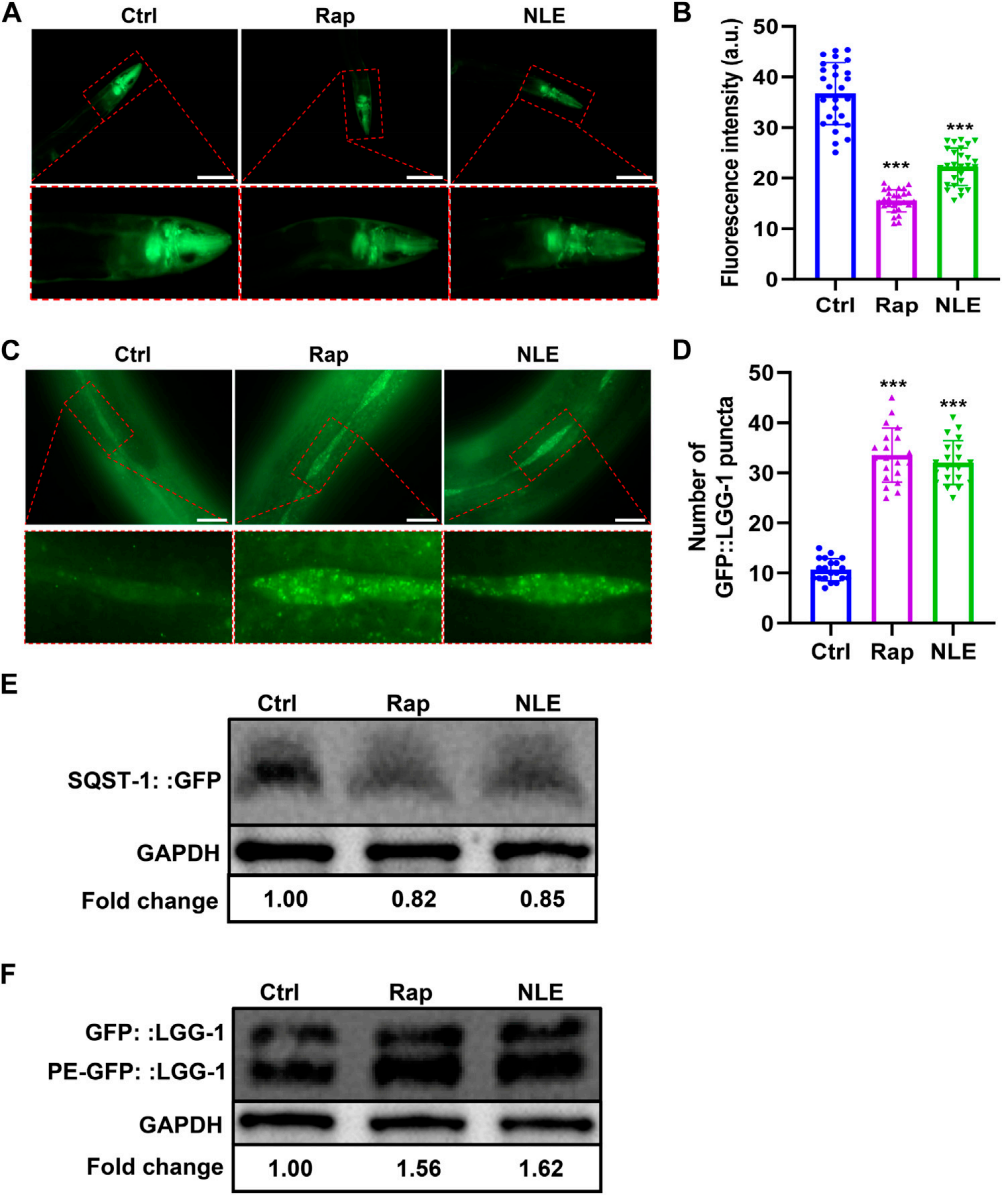
NLE induces autophagy in *Caenorhabditis elegans*. **(A,B)** Representative fluorescent images indicating the expression of SQST-1:GFP in the anterior head region of the BC12921 worms. The bar graphs show the quantification of green fluorescent protein (GFP) levels in **(A)**. The data are presented as mean ± SD (n = 20 nematodes per group). Scale bars: 200 μm. **(C,D)** Representative images showing the autophagosomes (GFP::LGG-1 puncta) in the seam cells of DA2123 worms. The bar graphs show the quantification of GFP::LGG-1 puncta. The data are presented as mean ± SD (n = 20 nematodes per group). Scale bars: 20 μm. **(E)** Representative Western blotting images indicating the protein levels of SQST-1:GFP in NLE-treated BC12921 worms, with the fold changes presented in the lower panel. Data are presented as mean ± SD (n = 3 independent experiments). **(F)** Representative Western blotting images indicating the ratio of PE-GFP::LGG-1 to GFP::LGG-1 in NLE-treated DA2123 worms, with the fold changes shown in the lower panel. The data are presented as mean ± SD (n = 3 independent experiments). For statistical analysis, the *p*-values were calculated using a two-tailed *t*-test. ***p ≤ 0.001. *p* < 0.05 was considered to be statistically significant.

The process of autophagy has garnered considerable attention in contemporary research, with studies underscoring its paramount significance in counteracting the adverse effects of aging and related diseases (Hughes et al., 2020). In this study, the potential of NLE to modulate autophagy was investigated using transgenic *C. elegans* models BC12921 and DA2123. The BC12921 model manifests a p62/SQST-1-GFP fusion protein, which diminishes during elevated autophagy activity (He et al., 2023). Fluorescence evaluations revealed a marked dip in GFP brightness (indicative of p62 protein amounts) in the NLE-administered BC12921 worms as opposed to the control group (Figures 4A,B). Moreover, Western blot analyses demonstrated that NLE decreased the protein levels of p62/SQST-1-GFP in BC12921 worms (Figure 4E), indicating degradation induced by NLE treatment. On the other hand, the DA2123 strain emits an LGG-1-GFP fusion protein that adheres to the autophagosome membrane, forming distinct structures upon triggering autophagy. The results in Figures 4C,D show that untreated DA2123 worms exude a spread-out fluorescence in various tissues, while the NLE treatment induces emergence of LGG-1^+^ structures specifically within their seam cells. Additionally, NLE elevated the ratio of phosphatidylethanolamine (PE) conjugated GFP::LGG-1 (PE-GFP::LGG-1) to non-lipidated GFP::LGG-1 in the DA2123 worms (Figure 4F). Collectively, these data demonstrate that NLE induces autophagy in *C. elegans*.

### 3.6 NLE counters Aβ-driven toxicity by activating autophagy in *C. elegans*

**FIGURE 5:**
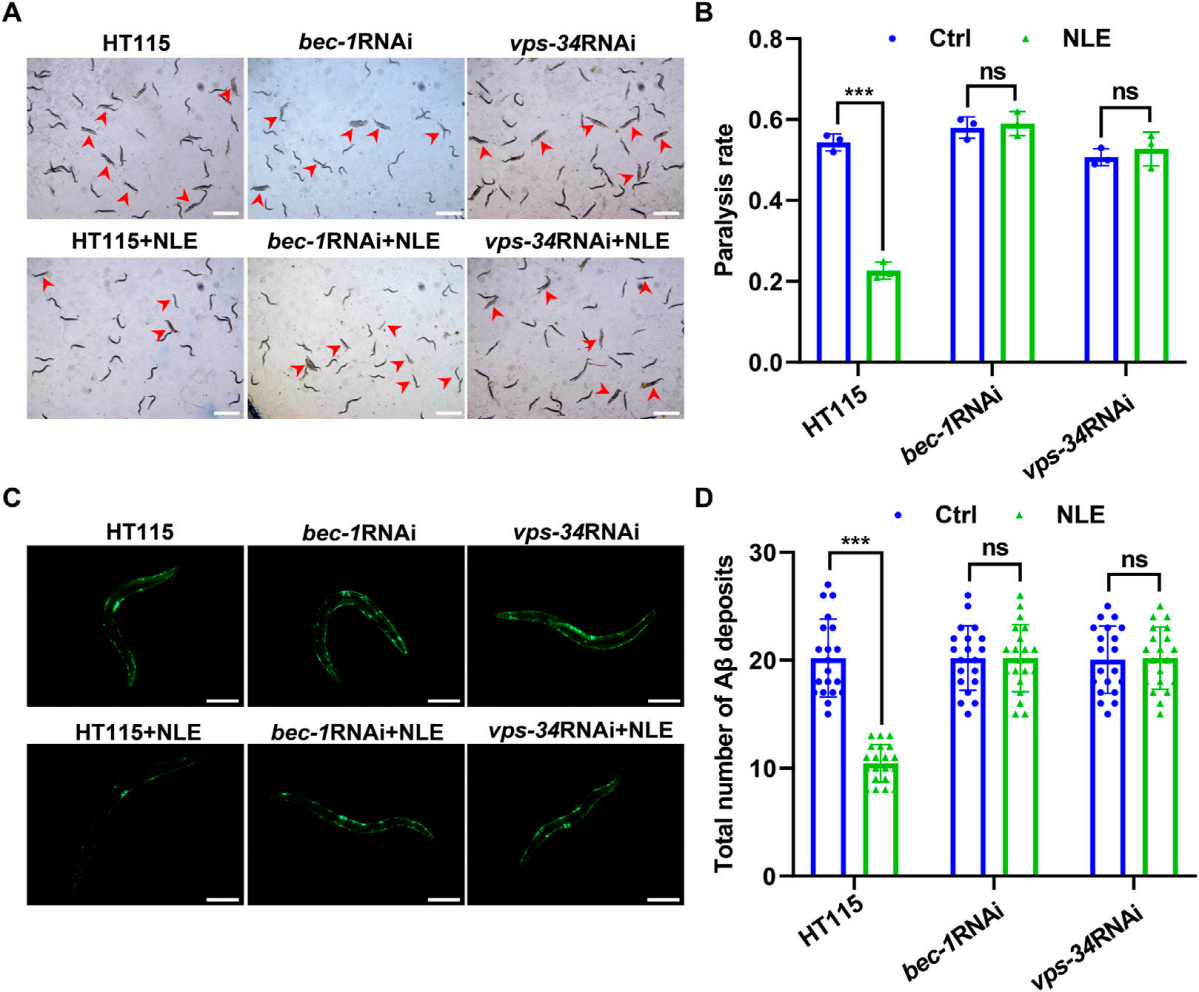
NLE mitigates Aβ-mediated pathology via autophagy genes in *Caenorhabditis elegans*. **(A,B)** Representative images showing the paralysis of CL4176 worms fed with RNAi bacteria having *bec-1* and *vps-34* genes. Scale bars: 1 mm. The bar graph indicates the paralysis rate of the worms. Data are presented as mean ± SD (n = 3 independent experiments). **(C,D)** Representative fluorescent images revealing Aβ deposits in CL2331 worms fed with RNAi bacteria having *bec-1* and *vps-34* genes. Scale bars: 200 μm. The bar graph quantifies the total Aβ deposits. The data are presented as mean ± SD (n = 20 nematodes per group). For statistical analysis, the *p*-values were calculated using a two-tailed *t*-test. ns *p* > 0.05 and ***p ≤ 0.001. *p* < 0.05 was considered to be statistically significant.

Considering the autophagy-inducing attributes of NLE in the *C. elegans* model organism, we examined whether the activation of autophagy could serve as a plausible mechanism to counteract the toxic effects induced by the Aβ protein. To achieve this, RNAi was employed as a highly effective gene silencing method in *C. elegans* to selectively inhibit two crucial genes integral to the autophagy process. Following the suppression of these genes, the extent of toxicity induced by Aβ in the AD models of *C. elegans* was evaluated. The results presented in Figures 5A,B indicate substantial alleviations in the paralysis rates in CL4176 worms when exposed to the control *E. coli* strain HT115 after NLE intervention. However, a marked contrast was observed when the worms consumed RNAi bacteria, leading to inhibition of *bec-1* and *vps-34* genes; under such conditions, the earlier reductions in the paralysis rates were conspicuously compromised. Simultaneously, the capability of NLE to mitigate the accumulation of Aβ aggregates was equivalently invalidated under the aforementioned conditions (Figures 5C,D). Together, the aforementioned findings provide compelling evidence suggesting a profound connection between the neuroprotective effects conferred by NLE and activation of the autophagy pathway in counterbalancing the toxic impacts mediated by Aβ in *C. elegans*.

## 4 Discussion

AD is primarily characterized by Aβ plaques and tau tangles and poses a growing global health threat that affects not only patients but also caregivers, families, and healthcare systems. Current treatments for AD only alleviate the symptoms, prompting search for other unconventional but long-term solutions (Ana et al., 2023). The natural compounds in medicinal and edible plants have garnered increasing attention, owing to their historical use in traditional medicine, multifaceted therapeutic potential, and excellent safety profiles (Ramalingum and Mahomoodally, 2014). *N. nucifera* has been a long-standing component of Asian traditional medicine and is renowned for its anti-inflammatory, antioxidant, and overall health-enhancing properties (Huang et al., 2010). This historical significance has prompted modern scientific investigations. In the present study, the therapeutic potential of NLE was assessed in the context of AD by utilizing the *C. elegans* model organism. The findings lay the groundwork for considering *N. nucifera* as a potential candidate for the management of AD.

The present research has yielded convincing evidence of NLE’s protective properties against AD. In the *C. elegans* models employed in this work, NLE effectively alleviates Aβ-induced paralysis, which is a key pathology of AD, with a notable impact and particularly at the concentration of 200 μg/mL. This not only supports but also emphasizes the potential of natural compounds like NLE and their diverse components to counteract the detrimental effects of Aβ aggregation in AD. The underlying mechanisms of NLE are also observed to be effective in inhibiting the formation and accumulation of Aβ aggregates, which are crucial precursors to AD pathology. The abnormal and uncontrolled aggregation of Aβ is a catalyst for intensified oxidative stress, a condition that leads to widespread neuronal dysfunction and subsequent cell death (Cheignon et al., 2018). The findings of this work reveal that NLE exhibits unique efficacy through a dual therapeutic mechanism that involves impeding the formation of Aβ aggregates and actively alleviating the oxidative stress induced thereof. This bimodal approach is essential for addressing the complexity of AD’s pathology. Additionally, our findings extend the scope of NLE’s protective nature. Following NLE treatment, *C. elegans* not only showed reduced paralysis but also exhibited extended lifespans and increased resilience to oxidative and thermal stresses; these indicate NLE’s potential for broader applications in promoting overall health, establishing it as a versatile protective agent.

Autophagy (derived from the Greek words “auto” meaning self and “phagy” meaning eat) is a highly conserved cellular mechanism responsible for degrading and recycling cellular components; it plays a crucial role in sustaining cellular homeostasis by eliminating misfolded proteins, damaged organelles, and other cellular debris, thereby ensuring cellular health and function (Aman et al., 2021). Disturbances in this process have been implicated in various pathologies, including neurodegenerative diseases like AD, where the accumulation of protein aggregates such as Aβ are hallmark features (Uddin et al., 2019). Consequently, strategies aimed at enhancing autophagic activities have shown promise in alleviating the symptoms of AD across diverse model organisms, including *C. elegans*, fruit flies, and mice (Deng et al., 2022). Currently, numerous compounds have been successfully identified for their abilities to enhance autophagy through the use of detection models. In *C. elegans*, two strains, namely DA2123 expressing LC3/LGG-1-GFP fusion protein and BC12921 expressing p62/SQST-1-GFP fusion protein, serve as valuable tools for assessing autophagy in response to various compounds (He et al., 2023). In addition, LC3-II, a marker for autophagic activation, was employed to monitor autophagy (Kuma et al., 2017). The present study showed that NLE increases the GFP-LGG puncta in DA2123 worms, thus providing evidence that NLE enhances autophagosome formation. However, an increase in autophagosome formation does not always signify normal autophagy flux. In certain pathological conditions, the accumulation of autophagic vacuoles and a corresponding increase in the LC3-II levels can be a result of impaired fusion between autophagosomes and lysosomes, leading to abnormal autophagy rather than autophagic activation (Yoshii and Mizushima, 2017). To comprehensively assess autophagy induction, the autophagy substrate p62 was examined in this work, and it was found to be effectively degraded, suggesting the presence of normal autophagic flow. This result indicates that autophagy indeed occurred, thereby dispelling concerns of autophagosome accumulation due to abnormal autophagy. Thus, the findings robustly support the conclusion that NLE is a potent inducer of autophagy. The significance of current findings is further underscored by the observed neuroprotective effects of NLE, particularly in alleviating Aβ-induced toxicity that is closely linked to its autophagy-activating capability. This direct association implies that NLE’s protective effects primarily result from the degradation and recycling of Aβ aggregates facilitated by the activated autophagy pathway. Considering these observations, it is proposed that NLE’s therapeutic potential in managing AD is fundamentally rooted in its ability to induce autophagy.

Although our findings in *C. elegans* show promising results, it is crucial to maintain a balanced perspective, given the complexities associated with the pathogenesis of AD in humans. The complexity and multifactorial nature of AD development and progression require careful and nuanced approaches when extrapolating results from model organisms to humans. *C. elegans* provides valuable insights owing to its similarities in conserved genetics and neuronal functions with humans (Culetto and Sattelle, 2000); however, it is important to recognize the inherent limitations associated with this model as they may not fully replicate the complete pathological landscape of AD observed in human patients. The complex interplay between the genetic, environmental, and lifestyle factors that contribute to the manifestation of AD in humans may not be fully captured in the *C. elegans* models, thus posing potential challenges when reflecting the full range of pathophysiological responses in humans. Taking these factors into account, it is critical to expand our investigation of the anti-AD efficacy and mechanisms of NLE to more complex model organisms, such as mice and zebrafish, to provide a more nuanced and comprehensive understanding of its potential therapeutic effects. Furthermore, it is essential to acknowledge the limitations to our single-dose experimental approach for dose-response assessment; although our study established the effectiveness of a 200 μg/mL dose of NLE in reducing paralysis in CL4176 worms, it is important to conduct further studies encompassing a broader range of doses to definitively establish the dose–response relationships in various assays. This will contribute to a more comprehensive understanding of the optimal dosage of NLE and its potential implications for AD management.

Identifying the key substances responsible for anti-AD activity is crucial for refining therapeutic strategies and optimizing clinical applications. Previous modern phytochemical studies have uncovered a diverse array of compounds in NLE, including alkaloids, flavonoids, lipids, glycosides, triterpenoids, vitamins, and carbohydrates. In our current investigation, we identified the principal compounds commonly found in NLE as nuciferine, leonticine, dauriciline, sinomenine, chrysoeriol, and kaempferol, which are in line with previous research (Pei et al., 2021). However, it is worth noting that the BPCs of NLE in the positive and negative ion modes differ slightly from those reported by Pei et al. (2021); this variation could stem from differences in the sample origin as well as analytical conditions and instrumentation utilized for the UHPLC-DAD-Q/TOF-MS/MS analyses. Additionally, some compounds in NLE have demonstrated potential anti-AD properties (Sharma et al., 2017). For instance, nuciferine has demonstrated the ability to restore AChE activities in both the blood and brain of alloxan-induced diabetic albino rats (Khan et al., 2022); phenolic compounds derived from NLE have also exhibited significant anti-AChE, anti-BChE, and anti-BACE-1 activities *in vitro* (Temviriyanukul et al., 2020). However, there is currently no literature on the role of NLE in promoting anti-AD effects through the activation of autophagy. Therefore, future research efforts could concentrate on investigating the individual compounds within NLE that could exert anti-AD effects by modulating the autophagy pathways.

## 5 Conclusion

This study demonstrates that NLE offers an intriguing therapeutic avenue for AD management through its ability to inhibit Aβ aggregation, ameliorate oxidative stress, and activate autophagy. Given the rich history of use of *N. nucifera* leaves in traditional medicine and their vast potential in modern pharmacology, further exploration and validation of NLE in more complex models along with elucidation of the specific active ingredients in NLE is expected to further improve its prospects for medical applications in age-related diseases, such as AD.

## Data Availability

The original contributions presented in the study are included in the article/Supplementary material; further inquiries can be directed to the corresponding authors.

## Abbreviations

AD: Alzheimer’s disease; Aβ: amyloid beta; NLE: *Nelumbo nucifera* leaf extract; *C. elegans*: *Caenorhabditis elegans*; Rap: rapamycin; CGC: *Caenorhabditis* Genetics Center; FUDR: 5-fluoro-2′-deoxyuridin; NGM: nematode growth medium; IPTG: isopropyl β-D-1-thiogalactopyranoside; SOD: superoxide dismutase; GPX: glutathione peroxidase, CAT: catalase; RNAi: RNA interference; SD: standard deviation; DHE: dihydroethidium.
